# Supplementary material for: Mathematical imaging methods for mitosis analysis in live-cell phase contrast microscopy
Source: Methods. 2017 Feb 15;115:91–9. doi: 10.1016/j.ymeth.2017.02.001 (PMC6414815; doi:10.1016/j.ymeth.2017.02.001)
Supplement: Supplementary file 1 [file mmc1.pdf]

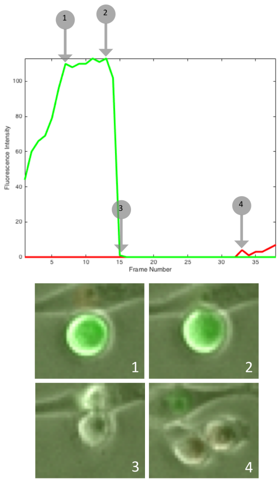

(a) Position 1 - DMSO control

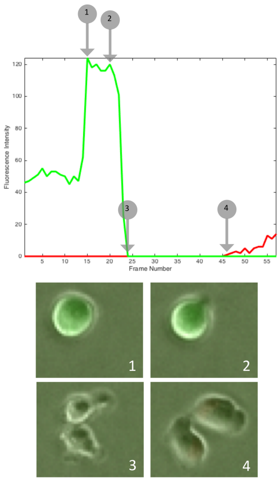

(b) Position 2 - DMSO control

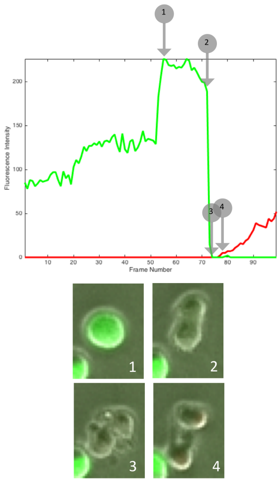

(c) Position 3 - DMSO control

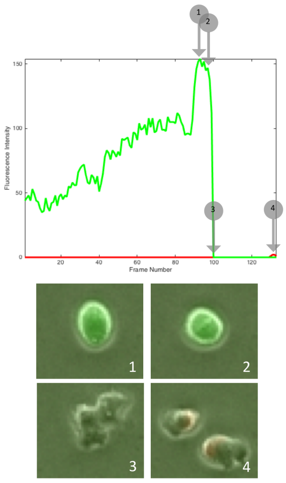

(d) Position 4 - 3nM paclitaxel

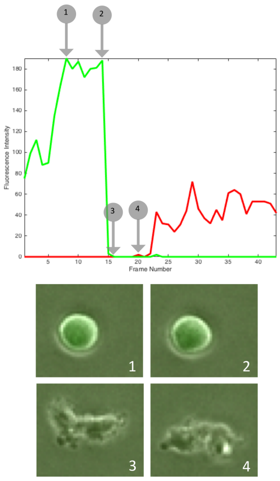

(e) Position 5 - 3nM paclitaxel

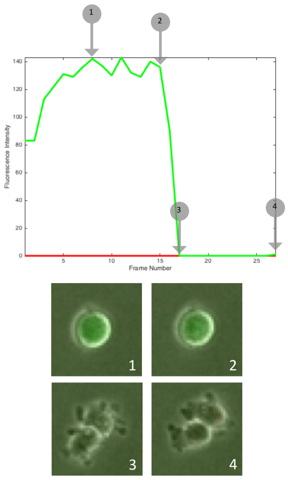

(f) Position 6 - 3nM paclitaxel

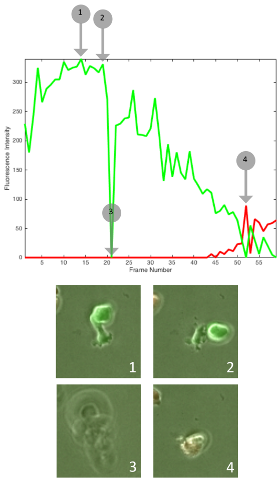

(g) Position 7 - 30nM paclitaxel

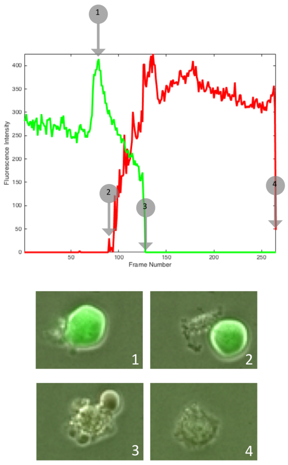

(h) Position 8 - 30nM paclitaxel

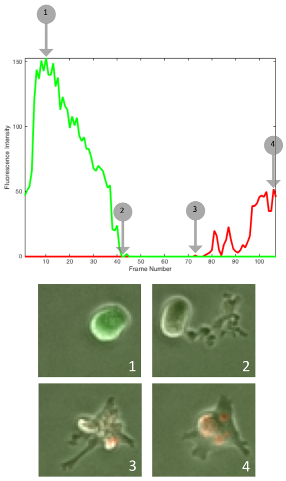

(i) Position 9 - 30nM paclitaxel

Supplementary Figure 1: Fluorescence intensity distributions. We can observe the red (CDT1) and green (Geminin) fluorescence intensity distributions for nine mitotic events over time, where the cell in (h) eventually dies. To be most accurate, they were obtained by manual analysis. The peaks of green fluorescence intensity indicate mitotic cells, which are about to divide. At this point in time, where circularity is maximal as well, cells are detected as being mitotic. The backwards tracking procedure stops as soon as the green fluorescent intensity drops significantly, which can be observed in the plots on the left hand sides of positions ”1”. After that, the forwards tracking procedure starts again where the mitotic cell has been detected and is stopped as soon as two daughter cells have been detected by means of the circular Hough transform or the green fluorescence intensity decreased below a small threshold, i.e. becomes almost zero. The drop of green intensity in position ”3” in (g) is due to an image acquisition artefact. Note that information provided by the green fluorescence intensity images is sufficient to define beginning and end of mitosis and that we do not use the red fluorescence intensity image sequences. In *MitosisAnalyser*, the green fluorescence images are pre-processed by the morphological operation of erosion and thresholding.

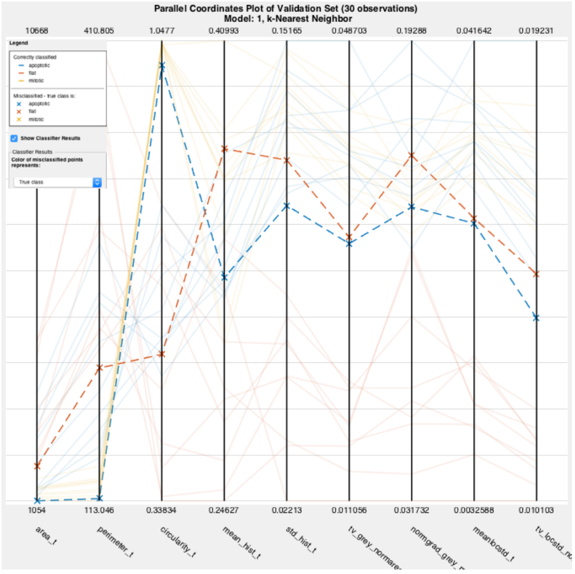

(a) Normalised parallel coordinates

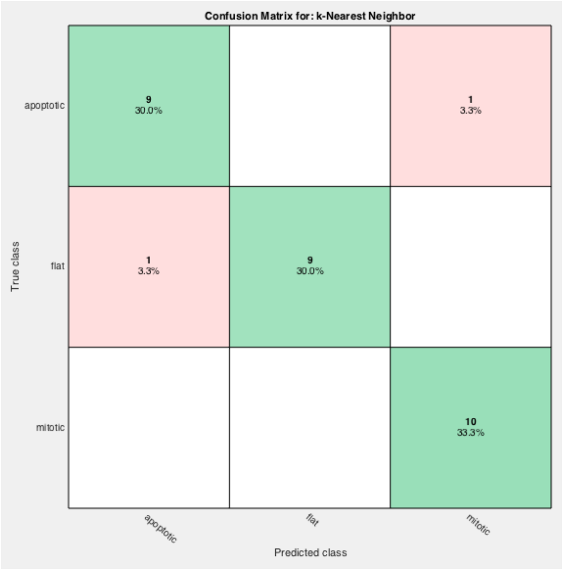

(b) Confusion matrix

Supplementary Figure 2: KNN classifier

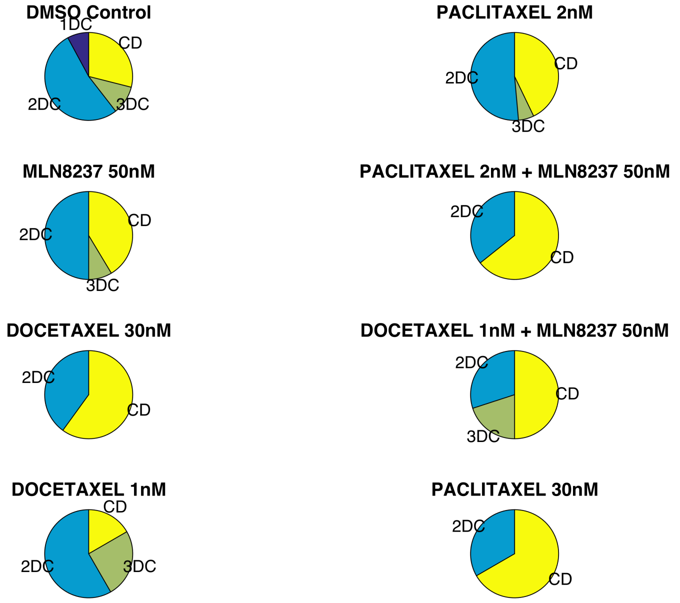

Supplementary Figure 3: Pie charts for fates of T24 cells treated with different drugs (1DC - 1 daughter cell, 2DC - 2 daughter cells, 3DC - 3 daughter cells, CD - cell death), where the number of analysed events is (from top left to bottom right): 38, 36, 58, 14, 5, 10, 12, 3

Parameter	Description	MIA PaCa-2	HeLa Aur A	T24
radiusMin	Minimum radius of mitotic cell	10	10	10
radiusMax	Maximum radius of mitotic cells	20	25	20
sensitivity	The higher, the more circular objects are detected	0.8	0.7	0.7
mitosisThreshold	Maximum mitosis duration	50	25	25
λ_1	Weight for normal velocity term inside of cell	1	0.5	5
λ_2	Weight for normal velocity term in the background	1	0.1	5
μ	Weight for length regularisation (smoothness)	10	8	17.5
ν	Weight for local standard deviation term	10	12	17.5
g_adj_low	Lower bound for rescaling of local standard deviation image	0.08	0.05	0.08
g_adj_high	Upper bound for rescaling of local standard deviation image	0.12	0.20	0.12
ω	Weight for area regularisation	1	1	10
timeStep	Time step Δt in gradient descent equation	1		
maxIterations	Maximum number of iterations for tracking contour evolution	5000	2500	5000
phiUpdate	Frequency of reinitialisation of level-set function with signed distance function	50	10	50
$\varepsilon_{\lvert\nabla\rvert \mathrm{Reg}}$	Parameter in regularisation of gradient magnitude	0.0001		
$\varepsilon_{\delta \mathrm{Reg}}$	Parameter in regularisation of Dirac delta function	2		

Supplementary Table 1: Mitosis detection and tracking parameters for different experiments

		MiA	CV	GAC
1	JSC	0.6103	**0.7056**	0.4960
	MHD	2.1713	**0.9120**	2.5202
2	JSC	0.5736	**0.6276**	0.3412
	MHD	3.1590	**1.3452**	4.8249
3	JSC	**0.6093**	0.4678	0.3561
	MHD	**2.1612**	3.0814	5.5271
4	JSC	**0.6741**	0.4493	0.2848
	MHD	**1.7944**	3.0597	6.5961
5	JSC	**0.4428**	0.4243	0.2608
	MHD	4.5354	**2.8466**	6.8695
6	JSC	**0.7133**	0.5030	0.3716
	MHD	**0.8024**	3.3760	6.0406
7	JSC	**0.6623**	0.3160	0.4835
	MHD	**1.6662**	4.3808	3.9340
8	JSC	**0.5402**	0.4014	0.4541
	MHD	3.5367	**3.2165**	4.6687
9	JSC	**0.5417**	0.1597	0.4175
	MHD	**2.0813**	6.5744	5.4570
10	JSC	**0.6877**	0.2445	0.4496
	MHD	**0.7584**	5.9506	4.7614
avg	JSC	**0.6050**	0.4299	0.3915
	MHD	**2.2666**	3.4743	5.1200

Supplementary Table 2: Quality measures JSC and MHD for segmentation of apoptotic cell images with *MitosisAnalyser* (MiA), the model by Chan and Vese (CV) and Geodesic Active Contours (GAC) in comparison with manual segmentation

Supplementary Algorithm 1 Mitosis Detection

Input: Image sequence, radiusMin, radiusMax, sensitivity, mitosisThreshold, distanceThreshold
  for every frame **do**
    Search for circularly shaped cells:
    [centres, radii, metrics] = `imfindcircles` (image, [radiusMin radiusMax], 'Sensitivity', sensitivity)
  end for
  Delete circles with centre coordinates that are identical or closer than the largest detected radius
  If applicable, compare to circles that have already been detected in previous frames:
  - number of frames to be checked specified by mitosisThreshold
  - maximum distance of centre coordinates specified by distanceThreshold
Output: Centres, radii, metrics, boundaries and frame numbers of detected mitotic cells

Supplementary Algorithm 2 Backwards Tracking

Input: Phase contrast images, [green fluorescent channel images,] initial contours, mitosisThreshold, λ_1, λ_2, μ, ν, ω, timeStep, maxIterations, phiUpdate
  Initialise level set function from circles surrounding mitotic cells
  while current frame within mitosisThreshold **and** circularity > threshold [**and** green intensity > threshold] **do**
    Reinitialise level set function by slightly increasing previous one
    Create region of interest around cell interior in order to increase speed
    Calculate function g
    while phi does not change significantly anymore or maxIterations reached **do**
      Update phi by gradient descent: $\phi^{\text{upd}} = \phi - \Delta t \cdot \delta_\varepsilon(\phi) \cdot \left(\mu\nabla \cdot \left(\frac{\nabla\phi}{|\nabla\phi|}\right) + \nu|\nabla\phi|\nabla \cdot \left(g\,\frac{\nabla\phi}{|\nabla\phi|}\right) + \omega P(\phi)\right)$
      $P(\phi) = \begin{cases} \int_\Omega (1 - H(\phi(x)))\;dx - t_{\text{area}}, & \int_\Omega (1 - H(\phi(x)))\;dx \leq t_{\text{area}}, \\ 0, & \text{otherwise} \end{cases}$
      Perform topology preservation combined with narrow band method
      Reinitialise phi every phiUpdate iterations
    end while
  end while
Output: Contours, statistics

Supplementary Algorithm 3 Forwards Tracking

Input: Phase contrast images, [green fluorescent channel images,] initial contours, radius, mitosisThreshold, λ_1, λ_2, μ, ν, ω, timeStep, maxIterations, phiUpdate
  while current frame within mitosisThreshold **and** outcome is unknown **do**
    Reinitialise level set function by slightly increasing previous one
    Create region of interest around cell interior in order to increase speed
    Calculate function g
    while phi does not change significantly anymore or maxIterations reached **do**
      Update phi by gradient descent (see Supplementary Algorithm 2)
      Perform topology preservation combined with narrow band method
      Reinitialise phi every phiUpdate iterations
    end while
    Apply `imfindcircles` again in a region of interest around the segmented cell
    Determine outcome based on detected circles and statistics / classification [and green fluorescent intensities]
  end while
Output: Contours, statistics, outcome
